# Formative Evaluation of the Central Coast Integrated Care Program (CCICP), NSW Australia

**DOI:** 10.5334/ijic.4633

**Published:** 2019-08-27

**Authors:** Hazel Dalton, Donna M. Y. Read, Angela Booth, David Perkins, Nick Goodwin, Anne Hendry, Tonelle Handley, Kate Davies, Michael Bishop, Rachael Sheather-Reid, Sarah Bradfield, Peter Lewis, Taryn Gazzard, Anthony Critchley, Sarah Wilcox

**Affiliations:** 1 Centre for Rural and Remote Mental Health, Faculty of Health and Medicine, University of Newcastle, New South Wales, AU; 2 Faculty of Health and Medicine, University of Newcastle, New South Wales, AU; 3 International Centre for Integrated Care, University of the West of Scotland, UK; 4 Central Coast Local Health District, NSW Health, New South Wales, AU

**Keywords:** formative evaluation, integrated care, Australia, case study, leadership, communication

## Abstract

**Introduction:**

Integrated care has been posited as an important strategy for overcoming service fragmentation problems and achieving the Quadruple Aim of health care. This paper describes the Central Coast Integrative Care Program (CCICP) a complex, multi-component intervention addressing 3 target populations and more than 40 sub-projects of different scale, priority and maturity. Details are provided of the implementation including activities undertaken for each target population, in the context of the Central Coast Local Health District (CCLHD) strategies and priorities. Key lessons are drawn from the formative evaluation.

**Methods:**

A mixed methods approach to the formative evaluation was taken. Key stakeholders, professional staff with an in-depth knowledge of the program, were invited to complete surveys (n = 27) and semi-structured interviews (n = 23). The evaluation employed co-design principles with dialogue between CCICP partners and researchers throughout the process and sought to achieve a shared understanding of the dynamic context of the program, and the barriers and enablers for the various interventions.

**Key lessons and conclusion:**

Seven interdependent key lessons have been identified. These distil down to the setting of clear objectives aligning with all the goals of partners, developing strong relationships, leadership at multiple levels and communication and the building of a common language.

## Introduction

The provision of health and care in Australia is fragmented, like other international healthcare systems, particularly in the United Kingdom and United States [1]. Services are delivered by multiple public and private providers, funded through a mix of federal and state governments and private entities with discrete goals and responsibilities. The Federal government supports primary care through patient fee subsidies for GP and allied health services, and locally commissioned primary care initiatives through Primary Health Networks (PHNs). The NSW state government funds Local Health Districts (LHDs) to deliver secondary and tertiary health care services in community and hospital settings.

Fee-for-service (market subsidy) arrangements operate in Australia, which reward the quantity of services rather than quality. These funding arrangements tend to overlook population health needs and discourage collaboration to meet user needs and achieve health outcomes. For example, primary care efficiencies may result in reduced hospitalisations which may, in turn, lead to hospital budget restrictions due to reduced activity. Thus perverse incentives apply which may result in unintended penalties for good practice [2].

As care provision and integrated care concepts extend beyond the boundaries of the health or ‘cure’ sector into social care and other sectors (aged care, youth services etc), it is worth describing the provision of social care in Australia [3]. Social services are provided through a series of groupings including aged care provision, family and community services, and the National Disability Insurance Scheme for those with lifelong disabilities. In these areas too, there are federal and state jurisdictional issues and responsibilities, as well as the commissioning of many community-based support services by non-government organisations.

Integrated care has been proposed as an important strategy for achieving the Quadruple Aim of health care. That is optimising health system performance through: improving the consumer’s experience; improving the health of populations; reducing per capita costs and improving the experience of the workforce [4]. Whilst the Australian system does very well compared to other OECD countries in relation to population health outcomes and the care process (including prevention, safety, consumer involvement and coordination of care) it ranks poorly in terms of equity [1]. Recognising the need to address health inequities as well as the rising costs of healthcare and increasing demands, the NSW Government drafted a state health plan. This plan aimed to improve the health of the NSW population equitably and sustainably by providing ‘the right care, in the right place, at the right time’. The key commitments of the plan are prevention, excellence in clinical care and integrated care [5].

To develop and progress integrated care, the Central Coast Local Health District (CCLHD) in partnership with public and private primary care health agencies became one of three NSW demonstrator sites in 2014 [6].

The appointment as a demonstrator site provided the CCLHD an opportunity, and resources, to test new ways of providing health care with the aim of better integration and improved outcomes for Central Coast residents. This opportunity aligned well with the CCLHD core Caring for the Coast Strategy [7]. The Central Coast Local Health District Demonstrator Project from here on will be referred to as the Central Coast Integrated Care Program (CCICP). This program was broad-ranging and ambitious, embracing the twin challenges of implementing integrated care and achieving large-scale system transformation, both of which are known to be difficult [8].

The Centre for Rural and Remote Mental Health was commissioned to conduct a formative evaluation of the CCICP during 2017, which sought to: describe the CCICP, examine component activities, assess their contribution to the goals, and consider evidence of system progress towards integrated care. The last of these objectives was achieved by examining perspectives of CCICP key stakeholders structured around the dimensions of Project INTEGRATE [9], an internationally validated framework designed to benchmark the progress of integrated care projects against criteria associated with successful adoption [10].

The purpose of this paper is to describe the formative evaluation of the CCICP, provide details on the implementation of program activities, including activities undertaken in each target group, in the context of CCLHD strategies and priorities. In addition, it describes key lessons that have come out of the formative evaluation of this multi-component program. Progress towards integrated care mapped to the Project INTEGRATE Framework are discussed in Read et al. [11].

## Central Coast Context

**Figure 1:**
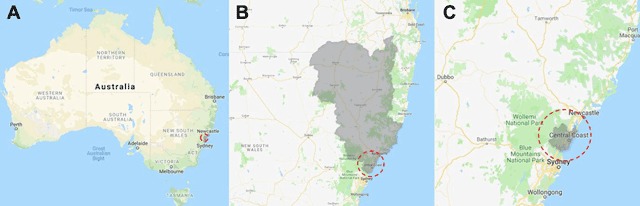
Map of Central Coast (red dashed circle), within New South Wales Australia (**A**), within the Hunter New England Central Coast Primary Health Network jurisdiction (**B**, grey shaded area) and the Central Coast Local Health District (**C**, grey shaded area).

The Central Coast of New South Wales Australia spans 1681km^2^ with 340,000 residents [12] (see Figure 1). The main health service issues and needs of the CCLHD were identified as:

- increasing attendances and admission rates in hospitals operating at, or over, capacity;
- increasing burden of chronic disease and obesity; and
- high proportions of aged, vulnerable young people and people living with chronic and complex conditions.

The Central Coast has a defined geography and service footprint, the agencies pertinent to the CCICP include CCLHD, Hunter New England Central Coast Primary Health Network (HNECCPHN), Yerin Aboriginal Health Service Inc., a well-established GP collaboration unit, the Department of Family and Community Services (FACS) and the Department of Education (DoE).

Prior to the demonstrator opportunity, the CCLHD had implemented several initiatives aimed at improved care integration including the Central Coast GP Collaboration Unit, the GP-Hospital Integration Project, and numerous successful partnerships of nurses in general practices (including diabetes education, shared midwifery, youth health clinics and mental health liaison). The GP collaboration unit is dedicated to working with general practices (private businesses) to progress and improve service models that connect with the local health service and primary health network (and its predecessor organisations).

The Central Coast area is outlined below (see Figure 1), in red dashed circle, within the context of NSW Australia (A), within the Hunter New England Central Coast Primary Health Network jurisdiction (B) and the Central Coast Local Health District (C).

## Description of Central Coast Integrated Care Program

As part of their proposal to be an integrated care demonstrator, the CCLHD agreed to undertake a number of activities to better integrate care (see **Box 1**). In line with NSW Health expectations for demonstrator sites, innovation and learning were adopted as underlying principles [6]. Initiatives were explored and trialled and their ability to augment integrated care outcomes in the Central Coast context was considered. This activity was conceived as formative work for a ten-year vision to transform the care system on the Central Coast.

**Box 1: Core objectives of the CCICP Implementation Plan**

- Developing a commissioning function jointly governed between the LHD and the then Central Coast NSW Medicare Local (now HNECC PHN), taking in a whole of system approach to the region’s health and social needs, working with stakeholders in prioritising target populations, service design, resource allocation and contracting.
- Enabling an integrated care system architecture that would be person-centred and allow movement towards anticipatory care for people with higher needs (away from system-initiated reactive care).
- Changing models of care for three key target population groups:
  - vulnerable young people,
  - vulnerable older people, and
  - people with chronic and complex conditions.

**Figure 2:**
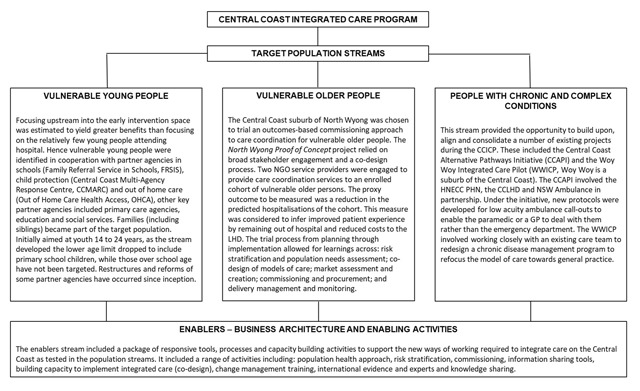
Central Coast Integrated Care Program Overview.

The CCICP is a complex, multifaceted intervention within three target populations and more than 40 sub-projects of different scale, priority and maturity have been undertaken thus far. Here we describe the activities undertaken within the target population streams and the underpinning enablers (Figure 2). Activities and preliminary outcomes (where available) for each stream are outlined. Business architecture and enabling activities have supported the overall process of integration (see Figure 2).

The CCICP team were co-located and comprised an operational leader who supervised four stream managers who either oversaw the implementation of one of the population streams or the enablers stream. These managers received central administration and project management support. This core team was assembled to work in collaboration with key partners in and outside the Central Coast LHD including HNECC PHN, the GP Collaboration Unit, FACS, NSW Ambulance, the NSW Agency for Clinical Innovation (ACI), Department of Education (DoE), and the Benevolent Society. A governance structure was established which included key stakeholders to guide and oversee the program.

## Methods

A formative evaluation, namely “a rigorous assessment process designed to identify potential and actual influences on the progress and effectiveness of implementation efforts” [13], was conducted using a mixed methods approach during 2017. The focus of the evaluation was to track and describe program implementation and assess progress towards integrated care.

Data collection included a review of 25 project documents, completion of 23 semi-structured interviews and 27 surveys (45% response rate) to assess progress towards integrated care. The CCICP core team identified documentation and key informants to complete surveys and interviews. The purposive sample included operational staff and others in leadership and executive sponsoring roles from the different program streams, key projects and the CCICP more generally. Hence, key informants included staff from the organisations listed in Table 1, as well as GPs, government and non-government agencies who took part in the delivery of the Program.

**Table 1:** Program stream project characteristics.

	Vulnerable Youth			Vulnerable Aged	Complex & Chronic Care	

	FRS in Schools	CCMARC	OHCHA	NWPOC	CCAPI	WWICCP

Full name	Family Referral Service in Schools	Central Coast Multi-Agency Response Centre	Out of Home Care Health Access	North Wyong Proof of Concept	Central Coast Alternate Pathways Initiative	Woy Woy Integrated Care Coordination Pilot
General description	An opportunity to develop Communities of Care around vulnerable families by creating an early intervention program, working upstream to impact health and social vulnerabilities.	The first NSW co-located multi-agency child protection information exchange and triage service.	Integrated multi-agency responses to assessment and management of the health needs of children and young people in out of home care	North Wyong Care coordination trial, uses NGO employed care coordination of vulnerable older person cohort, under novel outcomes based commissioning contracts, wherein reduction in unplanned hospital bed days are the target outcomes. Service is free to clients, with providers paid on outcomes.	NSW Ambulance Paramedics trained to implement low acuity protocols to manage alternate pathway referrals for appropriate patients.	Testing transition from the Chronic Disease Management Program (CDMP) to a model focused on General Practice
Objectives	To work with families, reduce their barriers to engaging with services and to prioritise actions that will support young people to engage with learning.	To define health’s role in interagency responses to child protection. Increase effective information exchange between health and Department of Family and Community Services (FACS). Support multiagency quality initiatives that enable early intervention responses for children at risk of significant harm (ROSH)	To better understand the pathways into out of home care, and identify opportunities for better-integrated service delivery To provide a better, integrated approach to health assessment and treatment of young people in this region.	To improve care coordination for enrolled cohort and reduce unplanned hospital bed days. To trial care coordination delivered by non-health providers To trial outcomes based funding model To keep older people healthy and at home for longer	To reduce unnecessary hospital transports of low acuity patients and to reduce ambulance turnaround time at hospitals.	To transition and improve care coordination for complex clients within the community
Date	October 2016–present	November 2015–to present	January–December 2016	January 2017–present, Commissioning cycle initiated July 2014 (needs assessment)	Proof-of-concept–January 2014. Paramedic training December 2015, June 2016	April 2016 to March 2017
Size	3 school learning communities–10,790–5 high schools, 13 primary schools, 2 providers	Central Coast, NSW population	>1000 young people (30–50 new to care each month)	440 patients, 4 general practices, 2 NGO providers	108 NSW Ambulance paramedics Central Coast NSW population	109 patients, 2 care coordinators, 8 general practices, 39 GPs
Target Population	Students and their families where there is an identified risk of disengagement from learning and school attendance.	Children and young people at risk of significant harm who live on the Central Coast, NSW	Children and young people in out of home care are a high-risk group for health and social care vulnerabilities. 30% Indigenous and 117 in kinship placements	North East Wyong region. People identified as having high health need, low socioeconomic status and ageing–likely to benefit from care coordination.	Patients assessed by qualified paramedics as suitable for alternative referral options do not require transport to the ED via ambulance.	Woy Woy, NSW chronic care population
Single point of referral	Yes (schools)	Yes	Yes	Not applicable, cohort identified by Central Coast LHD and referred to providers	Triage via telephone contact with the ambulance service	Identified through Central Coast LHD Connecting Care Program
Risk Stratification	Yes	Yes–ROSH screening tool used by FACS	Yes	Yes	Yes	Yes
Inclusion criteria	Primary and high school students and their families Geographically defined	Vulnerable families in the Central Coast region	Young people (0–18 years) in out of home care in the Central Coast region	Aged 65 or over 1 unplanned admission over the last year 2+ chronic conditions Geographically defined	NSW Ambulance patient transportations of triage categories 4/5	Identified through Central Coast LHD Connecting Care Program
Partners	Family Referral Service, Central Coast LHD, Department of Education (DoE), Local School Principals, HNECC PHN, FACS	CCLHD, FACS, DoE, The Benevolent Society, Family Referral Service	Central Coast LHD, HNECC PHN, FACS	HNECC PHN ADSSI Home Living Kincare Health Services	HNECC PHN Central Coast LHD NSW Ambulance	Central Coast LHD GPs
Community & Primary Care focus	Yes	Yes	Yes	Yes	Yes	Yes
Co-design	Yes	Yes	Yes, with respect to establishing three working groups for priority action.	Yes	Yes	No
Care-coordination	Yes	Limited to coordination of referrals	Yes	Yes	Limited to coordination of alternative referrals.	Yes
Key facilitators	Family Engagement Workers, local school Principals, finding alignments with partner agencies goals and frameworks to progress work (aligned values)	Colocation of multiagency staff with formal structured collaboration meetings for information exchange and quality improvement	Collaboration and strong leadership. Clear common goals defined by FACs and Health Policy objectives	Evidence-informed planning, Outcomes Based Commissioning cycle, strong leadership, NGO market appetite to undertake work	Cooperative patients, usual care providers or available GP practices	Early implementer of the state-wide redesign on CDMP
Key challenges	Adequate needs assessment of families and their cooperation, support from local school Principals, restricted ability to fully partner with HNECCPHN, identification of systemic gaps in services (e.g. under 12s mental health, housing and accommodation, services to support behavioural issues for students)	Rigorous quality improvement framework, consistent approach of adoption and monitoring of changes Unclear goals from outset	Formal and informal partnership agreements, framework design with partners State-based review (Their Futures Matter review) and reform overrode the activities, limiting ability to go forward with planned changes at the time.	New ways of working for NSW Health: contracts, procurement procedures (different to commissioning), time pressures, privacy and ethics concerns, sharing the risk between Central Coast LHD and providers, contracts based 100% on outcomes, restricted ability to fully partner with HNECC PHN	New way of working for NSW Ambulance paramedics, collaboration with patients, usual care providers or available GP practice, restricted ability to fully partner with HNECCPHN	Implementing new model of care within existing program and workforce with entrenched ways of working.

The evaluation employed co-design principles with dialogue between CCICP partners and researchers throughout the process and sought to achieve a shared understanding of the dynamic context of the program, and the barriers and enablers for the various interventions. Hence the co-design principles of inclusivity, respect, participation, and iteration with a focus on outcomes were followed [14]. However, due to time constraints it was not possible to involve consumers and carers. This included two workshops with the core CCICP team at project inception (for program understanding in context and over time, and verification of evaluation goals) and a third mid-way during the formative evaluation (including a review of interim survey results, a situational analysis of CCICP progress, and reflections on interim evaluation outcomes). Feedback, verification and validation of findings were sought from the CCICP team, with a minimum of four researchers in attendance at each workshop. The project review, coupled with observations by the core CCICP team (both in workshops and directly) enabled a structured description of the major sub-projects in each stream, using a framework modified from Wodchis et al. [15].

Research team members were experienced researchers with expertise in integrated care, qualitative methodologies, quantitative methodologies, service design and translational research. For further details of the methods and results of the progress made toward integrated care see Read et al. [11].

## Implementation of the Central Coast Integrated Care Program

**Figure 3:**
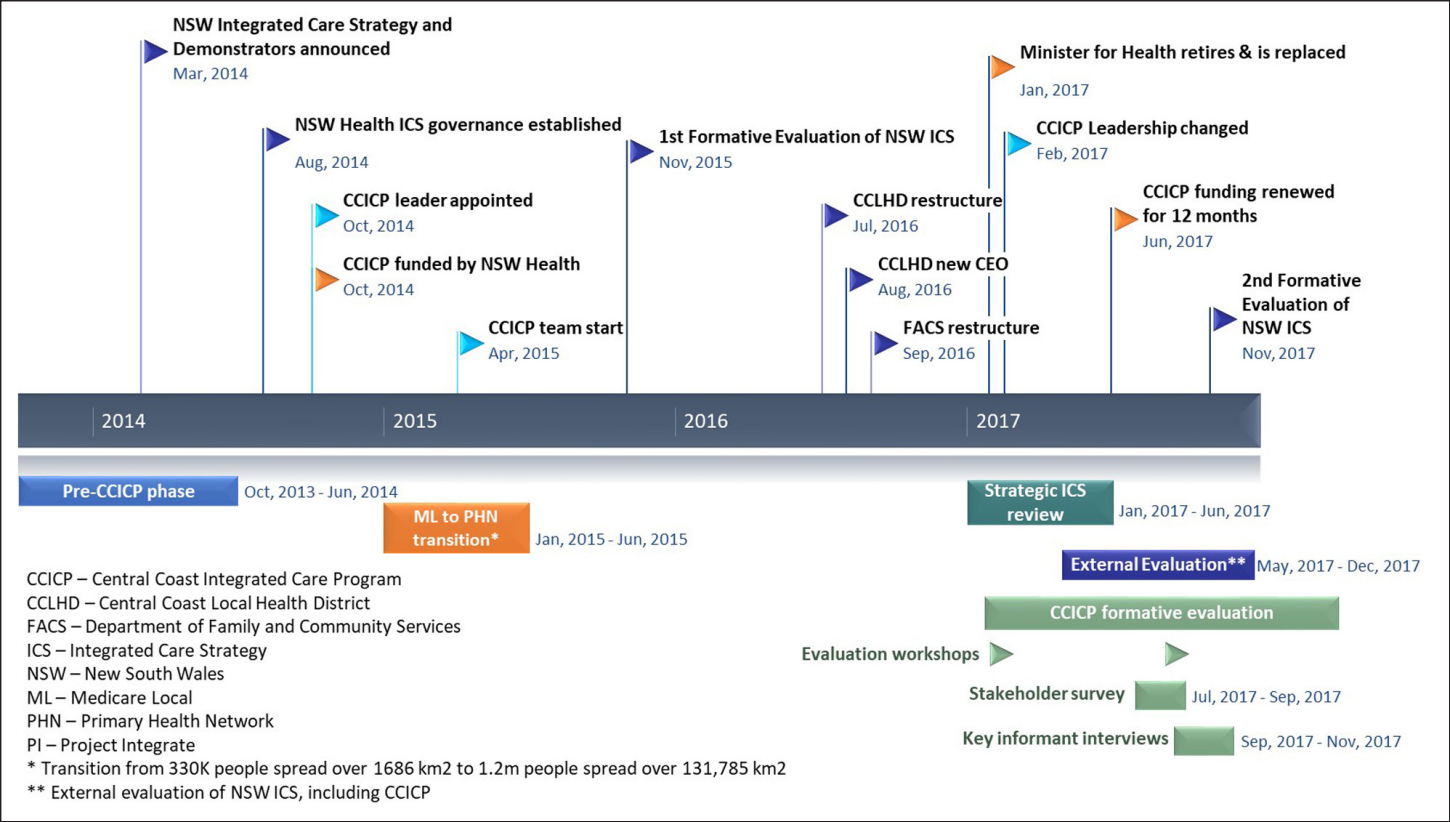
Key events and changes over the CCICP planning and implementation period.

The implementation context for the CCICP changed considerably over time with a range of unexpected and important events (see Figure 3). Contextual changes included changes both within the CCICP, the local health district and externally including staffing changes and restructures which affected program implementation and the pace of change. Most notably, the core CCICP team started six months after CCICP funding became available, which slowed momentum at a key early stage of implementation. Furthermore, a key partner in the original CCICP plan, the primary care organisation Medicare Local was restructured and emerged as a new entity, a Primary Health Network, with increased responsibilities in population (more than five-fold) and geography (more than 78-fold increase in km^2^) This significantly influenced planning and progress. The CCLHD (July 2016) and the social services agency Family and Community Services (FACS) (September 2016) underwent restructuring during the implementation window, affecting staffing mix and priorities.

Leadership changed at many levels during the project. The LHD Chief Executive Officer resigned and was replaced in August 2016, the state health minister retired and was replaced in January 2017 and the CCICP leader was seconded to another role in the local health district in February 2017. The timing of these changes was particularly challenging with the initial funding window finishing in mid-2017 and CCICP staff dependent on these funds, with a state-led strategic review of its integrated care strategy including demonstrators in the first half of 2017. The FACS leader changed with its restructure in September 2016 also. Thus momentum, confidence, innovation and partnered work are likely to have been impacted negatively.

However, some state policy changes and their resultant restructures, for example, changes to the protection of vulnerable children, have also led to an environment which is more conducive for state-based agency collaboration in provision of care for vulnerable youth.

Hence, contextual changes necessitated flexibility around the original CCICP plan. Nevertheless, original principles and strategies continued to inform the design of interventions. A population health approach with needs assessment emphasised the value of targeting streams and the chosen trial sites. Additionally, activities in each stream were tested. Characteristics of the major projects in each stream are summarised in Table 1 which shows a consistent approach to integrated care projects, clear objectives and target populations, referral mechanisms, risk stratification processes and inclusion criteria, external partners, community focus, co-design and care coordination.

The work to date, effectively two-years into a ten-year plan, has advanced progress towards the core objectives of the CCICP implementation plan (**Box 1**) which are consistent with the Caring for the Coast strategy. New working relationships have been established to enable a whole of system approach to health and social needs and some jointly commissioned initiatives. The integrated care architecture continues to be built with some progress, although further work is needed to establish true person-centred care [11]. Important advances have been made towards changing the models of care for the three target populations. That the objectives have not been fully achieved at this stage is unsurprising. As Wodchis et al. [15] note the changes needed for integrated care take time to achieve.

## Discussion - Lessons learned from the case

Many of the implementation lessons described below may apply to other client groups and services and may contribute to patient centred and integrated care when applied in combination.

### Set clear objectives, retain a core set of integrated care principles and allow flexible implementation

The CCICP has made clear progress on all of the core objectives described in **Box 1**. An Australia-first test of outcomes-based commissioning was trialled with vulnerable elderly people in North Wyong (see Table 1). Numerous enabling or infrastructure projects were completed to support particular projects and to provide system-wide support (see Figure 2); and new models of care for each of the three target populations were tested and implemented (see Figure 2).

The CCICP experienced considerable of contextual change throughout the implementation period including organisational and structural changes, changes of leadership at multiple levels, staff changes and new policies. These contextual changes influenced the day to day practical implementation of projects. Furthermore, numerous broad sub-projects which adopted a consistent approach to care model design informed by principles of integrated care (see Table 1) allowed learning to be shared across projects. Thus, a flexible approach which strongly emphasised objectives and principles ensured consistent models of care aligning with the original objectives.

### A tiered approach from low to higher risk-taking may enable more innovative projects to be attempted whilst maintaining support through early wins

The CCLHD set about an ambitious system-wide approach to transformational change over a ten-year period, based in a state-run health service. While the initial three years of demonstrator funding supported the initiation stage, the choice of populations and projects demonstrated a tiered risk approach in making changes that would facilitate integrated care provision. The program of work included varying levels of innovation to achieve both short-term and long-term dividends for service-users, the CCLHD and their partners who had varying levels of risk-tolerance.

The projects for people with complex and chronic conditions represented the safest choice and best chance of early wins by extending existing chronic disease management programs and increasing the proportion of care in primary care settings. This built upon existing programs and was also culturally acceptable. The next innovation was to select vulnerable populations rather than diseases or conditions, thereby, improving person-centred approaches and avoiding duplication of services. Low risk projects for the vulnerable older population were designed to reduce avoidable hospital admissions, so improving patient experiences and reducing costs to the CCLHD. The vulnerable young people stream was the most innovative and highest risk, investing in early interventions to promote healthy life-course trajectories in a target population who may need very expensive care in the future. Projects in the vulnerable youth stream involved close collaborations with partners beyond the traditional health service sphere, including education and social services, representing gains in horizontal care integration.

### Find alignment with the goals of partner agencies to increase buy-in and build sustainability

Working in partnership was a key feature of the approach taken within the CCICP; a focus on building relationships and increasing trust was evident. There was better progress in projects where the goals of multiple partners were strongly aligned. For instance, the Family Referral Service in Schools project was perceived to be contributing to key goal for a number of partners. For the CCLHD goals included improving access to care, early intervention and longer-term population health outcomes. For the HNECC PHN improving connections with GPs and allied health was important. For FACS improving access to social care services was a key goal while Education was concerned to increase and sustain engagement at school. Where the objectives of partners were clearly complementary there was evidence of stronger collaboration and increased buy-in by staff [16,17]. If projects are to progress and new services continue to develop beyond the pilot phase, interventions need to fit with the objectives held by multiple partners.

### Distribute leadership to retain and build shared vision and survive workforce turnover

The importance of leadership when implementing integrated care strategies is strongly supported in the literature [18,19,20,21]. However, this case demonstrated that in an ambitious transformation program like the CCICP, multiple levels of leadership with a clear understanding of program objectives and principles of integrated care is critical and must be supported by clear governance and a clear operating model.

Clear executive leadership at inception, with strong operational leadership, saw the program come to life, with further distribution of leadership through four stream managers who oversaw the target population and enablers projects. Implementation was well advanced, with the core team employed for more than a year, when the first changes of leadership occurred at the executive level in both the health district and social services (FACS). The strong operational leadership and momentum within the streams successfully engaged the new executive leadership and ensured continuing support for the CCICP. Six months later the CCICP operational leader moved to a different role in the organisation. This posed a significant challenge to the program, but by this time the program was strongly supported by the new health district CEO. One of the CCICP stream managers took on the operational leadership role, bringing experience, program knowledge and continuity to the program. However, the situation was further challenged by the appointment of a new health minister, the ending of special funding for demonstrators, and a strategic program review by the funding body (NSW Health). This series of changes was more difficult to navigate and uncertainty persisted amongst stakeholders about the future of the CCICP at the time of data collection. There were concerns that if integrated care was rushed from a special program (CCICP) into business-as-usual, the broader ambitions may be lost unless the new integrated care principles were firmly embedded across the service system. The executive leadership indicated ongoing support for integrated care and initiated discussions about a formal alliance between key partners, a future focus on building capacity, including a new research institute focussed on integrated care which is currently under construction.

### Document, learn and share lessons as you go

A feature of the CCICP was good knowledge sharing amongst the core CCICP team which was particularly enhanced by their co-supervision and colocation. However, the knowledge translation and mobilisation did not stop there, many issues papers were written and shared within the health district, other demonstrators and partners. These include assessments of the feasibility of risk stratification using General Practice data in NSW, issues of privacy and ethics of data sharing with partner agencies, and the views of stakeholders about their needs for shared care planning tools.

Also, lessons from earlier projects were transferred and applied to developing projects irrespective of the stream from which they arose. For example, the requirements analysis which had been generated for shared care planning was applied to a trial of a multi-agency web-based health and social care communication tool. This trial highlighted varied understandings and readiness amongst agencies to share information, which was taken into consideration in the trial of a customisable electronic patient education tool. The importance of this kind of reflexive practice has not been discussed in the literature before to our knowledge.

### Leverage your learning opportunities to increase engagement and rapport with partners and build a common language

For effective collaboration and to understand the distinct identities and roles of partners, trusting relationships [21] needed to be built. Whilst building key staff skills in both integrated care and change management, the CCICP deliberately maximised learning opportunities for relationship building. For example, partners from multiple agencies working on a project were brought together for change-management skills training. This was seen to have improved relationships significantly and ensured a common language amongst partners for the change management process. The importance of a common language among partners as they move into the unfamiliar territory of integrated care was recently discussed by Miller and Stein [22]. Furthermore, the CCICP sought opportunities to learn more about integrated care and facilitated local workshops with international experts that were attended by LHD and partners involved in the CCICP. At a narrower level, but with a significant impact, the process of evaluating the CCICP involved the core team and members of the executive, not simply the lead or a liaison person to facilitate contact. This collaborative approach led to lessons being articulated and acted upon in a more timely manner in a shared capacity.

### Communicate broadly to share what is working and expand the concept of integrated care

The CCICP developed a clear vision of integrated care on the Central Coast, they were able to share this well with executive leadership and partners. However, as time progressed, it was clear to the team that communication was crucial and they developed a formal communications plan during the evaluation period. There was a clear goal to build a shared understanding of integrated care since the concept varied greatly amongst stakeholders. The team found the Project INTEGRATE Framework [9] was helpful in articulating progress, scope and scale of activities, such that it allowed strengths, weaknesses and gaps to be identified in both the design, implementation and review phases.

### Reflections on the major difficulties and how learnings have been incorporated into current CCLHD management are outlined in Box 2

**Box 2: Key challenges and incorporated lessons from the Central Coast Integrated Care Program**

What were the major difficulties?

- The differences, and changes, in expectations between the Central Coast LHD and the State Government health department resulted in a misalignment of strategic objectives and financial incentives. The Local Health District had adopted a 10-year transformation plan and the State Government had more immediate objectives.
- Not surprisingly, following a series of changes in government ministers and staff the state government objectives changes towards the end of the funding period from a focus on experimentation and learning to an interest in summative achievements and accounting for the expenditure.
- The complexity of the CCICP interventions including the cessation of ineffective elements and the development of new interventions ‘mid-stream’ did not fit well with a government need for an overall summative assessment of the three very different demonstrator investments.

What learnings have been incorporated in current CCLHD management?

- The CCLHD has moved form an understanding of integrated care within a special project and special funding arrangement to an understanding that integrated care is to be understood as normal practice and business as usual.
- A senior integrated care manager has been appointed but key project staff have been appointed to strategically important positions across the District and are able to apply their special expertise within the key operation systems of the service.
- The District has built on its evolving partnerships with the University of Newcastle, The Hunter New England Health District and the Hunter New England Central Coast Primary Health Network to create a local research institute focusing on integrated care and population health which will support organizational learning and capacity building in a more comprehensive fashion building on the work of the formative evaluation reported in this paper.

## Conclusion

We have described the activities of the Central Coast Integrated Care Program. At the outset, the transformation of Central Coast LHD services from a fragmented and provider focus to an integrated, person- and population-focused approach understood to be a ten-year task. In considering the approaches undertaken by the CCICP, it is clear that the higher order goals have been maintained, even though the granular activities may have changed in response to contextual changes, reflective review, lessons learned and iterative improvement and planning. As discussed, this is still a young project that cannot be expected to have achieved fully its objectives.

Nevertheless, seven lessons likely to be of general interest have been identified. The formative evaluation highlighted a number of areas for attention. These can be summarised as: setting clear objectives which align strongly with partner goals, relationship building, leadership and communication. All of these are interdependent.

Trusting relationships do not happen without good communication, good leadership and clear objectives. Clear objectives aligned with core principles enabled the CCICP to navigate the contextual changes it faced flexibly, and to continue to build alliances with the Primary Health Network. Ensuring that project objectives met the needs of all partners ensured their continued participation, and continues in projects such as the Family Referral Service in Schools program. The case of the CCICP shows the wisdom of ensuring leadership is distributed and not limited to a single person has enabled the CCICP to traverse the inevitable changes in personnel. Communication, including training to build a common language, developing relationships and monitoring and assessing progress was also found to be an important feature of the gains made by the CCICP. It is likely these aspects of communication were facilitated by co-supervision and co-location of the CCICP team. This assessment and documenting of progress fed into the ambitious tiered system-wide approach attempted. The knowledge translation and learning capacity is being reinforced through the partnered investment of a research institute focussed on integrated care and the appointment of its inaugural director.

The CCICP program of work that has demonstrated the CCLHD’s capacity to design, develop and implement innovative models of care in conjunction with partner agencies that are underpinned by a core set of integrated care based principles.

## References

[1] Schneider, E, Sarnak, D, Squires, D, Shah, A and Doty, M. Mirror, mirror 2017: International comparison reflects flaws and opportunities for better US health care. 2017 [cited August 2018]. Available from: https://www.commonwealthfund.org/publications/fund-reports/2017/jul/mirror-mirror-2017-international-comparison-reflects-flaws-and.

[2] Cashin, C, Chi, Y-L, Smith, PC, Borowitz, M and Thomson, S. Health provider P4P and strategic health purchasing In: Cashin, C, Chi, Y-L, Smith, P, Borowitz, M and Thomson, S (eds.), Paying for performance in health care: Implications for health system performance and accountability. Maidenhead, UK: Open University Press, Mc-Graw-Hill Education; 2014.

[3] Goodwin, N. Understanding integrated care. International Journal of Integrated Care, 2016; 16(4). DOI: 10.5334/ijic.2530 PMC5354214 28316546

[4] Bodenheimer, T and Sinsky, C. From triple to quadruple aim: care of the patient requires care of the provider. The Annals of Family Medicine, 2014; 12(6): 573–576. DOI: 10.1370/afm.1713 25384822 PMC4226781

[5] NSW Health. NSW State Health Plan: towards 2021 North Sydney: NSW Ministry of Health 2014 [cited August 2018]. Available from: http://www.health.nsw.gov.au/statehealthplan/Publications/NSW-state-health-plan-towards-2021.pdf.

[6] NSW Health. NSW Integrated Care Strategy. 2016 [cited August 2018]. Available from: https://www.health.nsw.gov.au/integratedcare/Documents/ic-mef-2016.pdf.

[7] NSW Government. Caring for the Coast: Clinical Services Plan 2012–2022 Central Coast Local Health District (ed.). Gosford, NSW: Central Coast Local Health District; 2013 [cited September 2018]. Available from: http://www.cclhd.health.nsw.gov.au/wp-content/uploads/CCLHDClinicalServicesPlan2012-2022.pdf.

[8] Central Coast Local Health District. Caring for the Coast. n.d. http://www.cclhd.health.nsw.gov.au/caringforthecoast.

[9] Calciolari, S, González, L, Goodwin, N and Stein, V. The Project INTEGRATE Framework, EU Grant Agreement 305821. 2016 [cited September 2018]. Available from: from: http://www.projectintegrate.eu.com/wp-content/uploads/2017/04/The-Project-Integrate-Framework-TOP.pdf.

[10] Cash-Gibson, L and Rosenmoller, M. Project INTEGRATE–a common methodological approach to understand integrated health care in Europe. International Journal of Integrated Care, 2014; 14: e035 DOI: 10.5334/ijic.1980 25550690 PMC4276036

[11] Read, DMY, Dalton, H, Booth, A, Goodwin, N, Hendry, A and Perkins, D. Using the Project INTEGRATE Framework in Practice in Central Coast, Australia. International Journal of Integrated Care, 2019; 19(2): 10 Published 2019 Jun 21. DOI: 10.5334/ijic.4624 PMC6588025 31244564

[12] Australian Bureau of Statistics. Central Coast (SA4) (102) 2017 http://stat.abs.gov.au/itt/r.jsp?RegionSummary&region=102&dataset=ABS_REGIONAL_ASGS&geoconcept=REGION&datasetASGS=ABS_REGIONAL_ASGS&datasetLGA=ABS_NRP9_LGA&regionLGA=REGION&regionASGS=REGION.

[13] Stetler, C, Legro, M, Wallace, C, Bowman, C, Guihan, M and Hagedorn, H, et al. The Role of Formative Evaluation in Implementation Research and the QUERI Experience. Journal of General Internal Medicine, 2006; 21(S2): S1–S8. DOI: 10.1111/j.1525-1497.2006.00355.x PMC2557128 16637954

[14] NSW Council of Social Service. Principles of Co-design. 2016 [cited June 2019]. Available from: https://www.ncoss.org.au/sites/default/files/public/resources/Codesign%20principles.pdf.

[15] Wodchis, W, Dixon, A, Anderson, G and Goodwin, N. Integrating care for older people with complex needs: key insights and lessons from a seven-country cross-case analysis. International Journal of Integrated Care, 2015; 15(6). DOI: 10.1111/j.1525-1497.2006.00355.x PMC4628509 26528096

[16] Johnson, P, Wistow, G, Schulz, R and Hardy, B. Interagency and interprofessional collaboration in community care: the interdependence of structures and values. Journal of Interprofessional care, 2003; 17(1): 70–83. DOI: 10.1080/1356182021000044166 12772471

[17] Mitchell, SM and Shortell, SM. The governance and management of effective community health partnerships: a typology for research, policy, and practice. The Milbank Quarterly, 2000; 78(2): 241–289. DOI: 10.1111/1468-0009.00170 10934994 PMC2751154

[18] Amelung, V, Chase, D and Reichert, A. Leadership in Integrated Care In: Amelung, V, Stein, V, Goodwin, N, Balicer, R, Nolte, E and Suter, E (eds.), Handbook Integrated Care. Cham, Switzerland: Springer International Publishing; 2017 p. 221–236. DOI: 10.1007/978-3-319-56103-5_14

[19] Borgermans, L, Marchal, Y, Busetto, L, Kalseth, J, Kasteng, F, Suija, K, et al. How to improve integrated care for people with chronic conditions: Key findings from EU FP-7 Project INTEGRATE and beyond. International Journal of Integrated Care, 2017; 17(4). DOI: DOI: 10.5334/ijic.3096 PMC5854097 29588630

[20] Dolan, B, Gullery, C, Hamilton, G and Meates, D. New Zealand: Canterbury Tales In: Amelung, V, Stein, V, Goodwin, N, Balicer, R, Nolte, E and Suter, E (eds.), Handbook integrated care. Gewerbestrasse 11, 6330 Cham, Switzerland: Springer International Publishing AG; 2017 DOI: 10.1007/978-3-319-56103-5_36

[21] Kirst, M, Im, J, Burns, T, Baker, GR, Goldhar, J, O’campo, P, et al. What works in implementation of integrated care programs for older adults with complex needs? A realist review. International Journal for Quality in Health Care, 2017; 29(5): 612–624. DOI: 10.1093/intqhc/mzx095 28992156 PMC5890872

[22] Miller, R and Stein, KV. Building Competencies for Integrated Care: Defining the Landscape. International Journal of Integrated Care, 2018; 17(6). DOI: 10.5334/ijic.3946

